# Integrating No.3 lymph nodes and primary tumor radiomics to predict lymph node metastasis in T1-2 gastric cancer

**DOI:** 10.1186/s12880-021-00587-3

**Published:** 2021-03-23

**Authors:** Xiaoxiao Wang, Cong Li, Mengjie Fang, Liwen Zhang, Lianzhen Zhong, Di Dong, Jie Tian, Xiuhong Shan

**Affiliations:** 1 Department of Radiology, Affiliated People’s Hospital of JiangSu University, Zhenjiang, People’s Republic of China; 2 CAS Key Laboratory of Molecular Imaging, Beijing Key Laboratory of Molecular Imaging, The State Key Laboratory of Management and Control for Complex Systems, Institute of Automation, Chinese Academy of Sciences, Beijing, People’s Republic of China; 3 School of Artificial Intelligence, University of Chinese Academy of Sciences, Beijing, People’s Republic of China; 4 Beijing Advanced Innovation Center for Big Data-Based Precision Medicine, School of Medicine, Beihang University, Beijing, People’s Republic of China; 5 Engineering Research Center of Molecular and Neuro Imaging of Ministry of Education, School of Life Science and Technology, Xidian University, Xi’an, People’s Republic of China; 6 Zhuhai Precision Medical Center, Zhuhai People’s Hospital (Affiliated With Jinan University), Zhuhai, People’s Republic of China

**Keywords:** Stomach cancer, Lymph nodes, Nomogram

## Abstract

**Background:**

This study aimed to develope and validate a radiomics nomogram by integrating the quantitative radiomics characteristics of No.3 lymph nodes (LNs) and primary tumors to better predict preoperative lymph node metastasis (LNM) in T1-2 gastric cancer (GC) patients.

**Methods:**

A total of 159 T1-2 GC patients who had undergone surgery with lymphadenectomy between March 2012 and November 2017 were retrospectively collected and divided into a training cohort (n = 80) and a testing cohort (n = 79). Radiomic features were extracted from both tumor region and No. 3 station LNs based on computed tomography (CT) images per patient. Then, key features were selected using minimum redundancy maximum relevance algorithm and fed into two radiomic signatures, respectively. Meanwhile, the predictive performance of clinical risk factors was studied. Finally, a nomogram was built by merging radiomic signatures and clinical risk factors and evaluated by the area under the receiver operator characteristic curve (AUC) as well as decision curve.

**Results:**

Two radiomic signatures, reflecting phenotypes of the tumor and LNs respectively, were significantly associated with LN metastasis. A nomogram incorporating two radiomic signatures and CT-reported LN metastasis status showed good discrimination of LN metastasis in both the training cohort (AUC 0.915; 95% confidence interval [CI] 0.832–0.998) and testing cohort (AUC 0.908; 95% CI 0.814–1.000). The decision curve also indicated its potential clinical usefulness.

**Conclusions:**

The nomogram received favorable predictive accuracy in predicting No.3 LNM in T1-2 GC, and the nomogram showed positive role in predicting LNM in No.4 LNs. The nomogram may be used to predict LNM in T1-2 GC and could assist the choice of therapy.

**Supplementary Information:**

The online version contains supplementary material available at 10.1186/s12880-021-00587-3.

## Background

Gastric Cancer (GC) is rampant around the world, especially in East Asia [1]. Surgery is the primary treatment for patients with early gastric cancer (EGC), however, a number of sequelae (indigestion, iron deficiency, etc*.*) can seriously reduce the patient’s quality of life for open surgery [2]. In order to improve the prognosis, less invasive surgical alternatives, such as endoscopic submucosal dissection and endoscopic mucosal resection, are used for the treatment of EGC [3]. However, endoscopic resection is only considered for tumors with a low risk of lymph node metastasis (LNM) [4].

Studies have found that LNM exist in 8.2–19.7% EGC [5–8]. Sentinel lymph node (SLN) biopsy, an invasive method, was used for detection of metastatic LNs in GC [9]. SLN biopsy was a promising tool to assess the LNM in T1-2 GC patients [10–12]. However, there are still debates regarding the effectiveness of LN detection techniques and oncological safety of biopsy. Kitagawa et al. [13] and Miyashiro et al. [14] used different SLN biopsy methods, but consequently obtained different false-negative rates (7% and 46.4%, respectively). Besides, noninvasive medical imaging like CT is routinely used to assess perigastric LNs. However, the accuracy of CT detection of LN in early GC is approximately 60%, which is unsatisfactory [15]. At present, it is still unable to accurately predict LNM of EGC preoperatively.

In recent years, Artificial Intelligence (AI) has been widely used in the field of medicine. Machine Learning (ML)-based tools have been used in Prediction of LNM, Risk assessment of cancer, lesion detection, staging, evaluation of prognosis and curative effect analysis. Radiomics based on this technology may improve the tumor patient management, screening strategy and customized treatment plan [16–22]. Studies have shown that radiomics, the technique of converting medical images into mineable data and high-dimensional features, has been proven to improve diagnostic and prognostic accuracy in oncology [23–26]. It had been widely applied to the prediction of LN metastasis in GC, colorectal cancer and occult peritoneal metastasis in advanced GC and achieved satisfactory results [27–35].

At present, the published radiomics research in finding the predictors of LNM mainly use tumor radiomics characteristics or other characteristics related to patients. However, the ability to accurately predict LNM may be affected by relying solely on the radiomics characteristics of primary tumors [33]. Thus, this study aimed to predict preoperative LNM in T1-2 GC patients by integrating the radiomics characteristics of LN and primary tumors. The LNs of the stomach are given station numbers as No.1–No.16 [36, 37]. Researches showed that the incidence rate of LNM in No.3 station was the highest (No.1, 2.5%; No.2, 4.8%; No.3, 11.6%; No.4, 6.5%; No.5, 0.5%; No.6, 7.6%) in EGC [38–40]. Therefore, by integrating the quantitative radiomics characteristics of No.3 LNs and primary tumors, we developed and validated a radiomics nomogram to better predict preoperative LNM in patients with T1-2GC.

## Methods

### Patients

The Institutional Review Board of our hospital approved this retrospective study and the requirement for informed consent was waived.

The inclusion criteria for the training and testing cohorts were as follows: (a) patients who underwent surgery with curative intent for T1-2 GC and with pathological results; (b) LN dissection performed; (c) excisional LN with detailed grouping and pathological diagnosis; (d) standard contrast-enhanced CT performed less than 10 days before surgical resection. The exclusion criteria were: (a) hypotensive drug taboo (such as glaucoma, prostatic hypertrophy, etc.); (b) preoperative therapy (radiotherapy, chemotherapy, or chemoradiotherapy); (c) concurrent with other tumors or diseases; (d) patients with variation of the left gastric artery; (e) invisible lesions on CT images.

A total of 159 patients between March 2012 and November 2017 were enrolled in this study (113 males, 46 females; average age, 61.78 ± 10.47 years). All the patients were randomly divided into two independent cohorts: a training cohort, containing 80 patients (53 males, 27 females; average age, 61.78 ± 11.11 years), and a testing cohort, containing 79 patients (60 males, 19 females; average age, 61.78 ± 9.77 years).

Clinical data, including gender, age, carcinoembryonic antigen (CEA: 0–5 ng/mL), carbohydrate antigen 19-9 (CA19-9: 0–40 03B u/mL), cancer antigen 125 (CA125: 0–35 u/mL), pathologic grade (see detailed description in Additional file 1: Table S1), CT-reported LN metastasis status from radiologist, and tumor infiltration depth, were obtained by reviewing the medical records.

### CT data acquisition

All patients fasted for at least 4 h, and 20 mg anisodamine (654-2) was administered intramuscularly to reduce gastrointestinal peristalsis 10 min prior to CT examination. 800–1000 mL warm water was drank to distend the stomach. CT was performed using a 256-Slice (Brilliance iCT, ROYAL PHILIPS, Eindhoven, Netherlands) or a 64-slice (SOMATON sensation64, SIEMENS Healthineers, Muenchen, Germany) multi-slice spiral CT. Patients underwent both unenhanced and two-phase enhanced CT examinations (arterial phase: 35 s after injection; venous phase: 70 s after injection). The CT scans, covering the entire stomach region, were acquired during a breath-hold with the patient supine in all of the phases. During the enhanced CT scan, patients were infused with 1.5 mL/kg of the non-ionic contrast material (iohexol, Yangzi River Pharmaceutical Group, Jiangsu, China; iIodine concentration: 300 mg/mL) with a pump injector (Ulrich CT Plus 150, Ulrich Medical, Ulm, Germany) at a rate of 3.0 mL/s into the antecubital vein. The imaging parameters were as follows: 120 kV; 220–250 mAs; rotation time: 0.5 s; detector collimation: 128 × 0.625 mm or 32 × 0.6 mm; field of view: 400 × 400 mm; matrix: 512 × 512; reconstruction slice thickness: 5 mm for axial plane, and 3 mm for coronal and sagittal plane.

### Pipeline

**Fig. 1:**
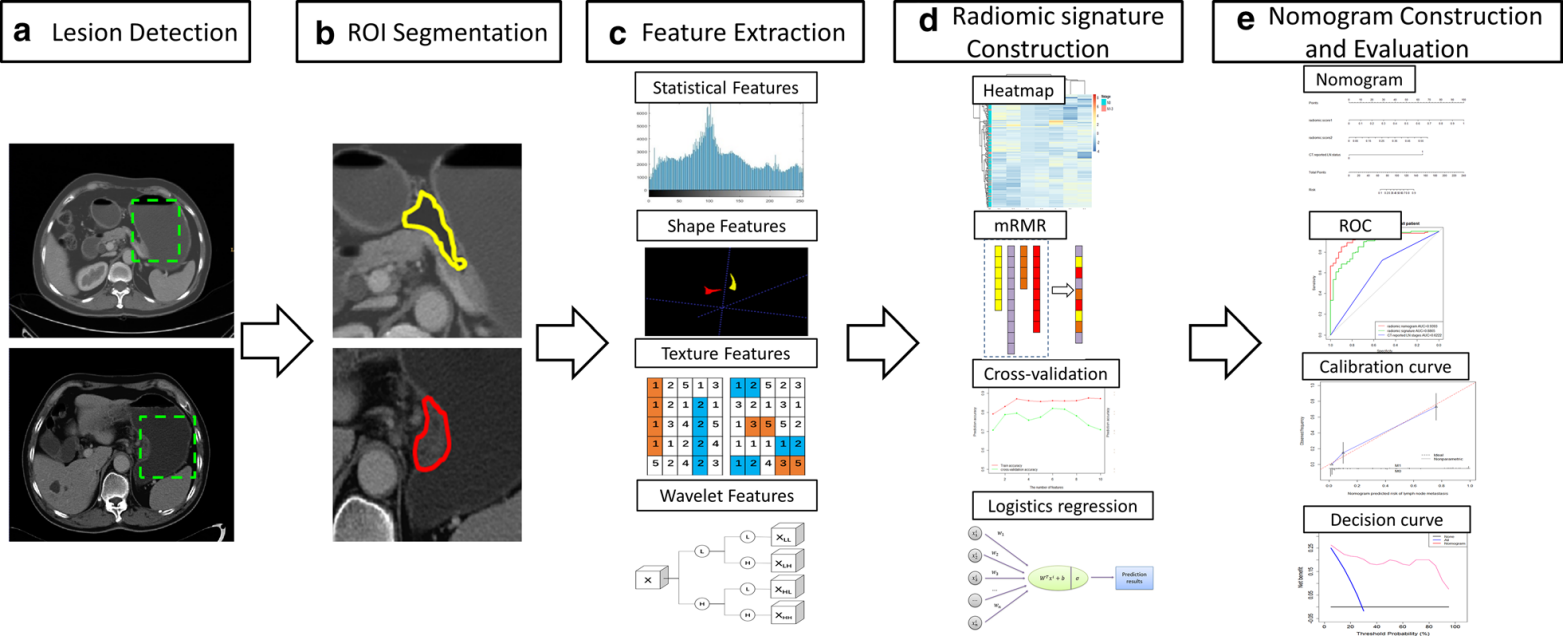
Radiomic workflow in this study

The pipeline of this study includes five steps: lesion detection, region of interest (ROI) segmentation, radiomic feature extraction, radiomic signature building, and nomogram construction and evaluation (Fig. 1).

### Detection of lesion on CT images

All CT images were reviewed by a radiologist with more than 10 years of experience in GC diagnosis. Localization of GC lesions: the 159 patients selected in this study all had the results of gastroscopy and CT examination. Combined with gastroscopy and CT images (axial, coronal and sagittal images), the lesions could be located. The diagnostic criteria of CT-reported LN metastasis-positive were shown as follows: short-axis diameter of LN ≥ 5 mm, the ratio of short diameter to long diameter of LN ≥ 0.7, and the plain CT value of LN ≥ 25 HU or venous phase CT value of LN ≥ 75 HU; or multiple LNs were fused together even if above conditions were not satisfied.

### ROI segmentation on CT images

Two 2-dimensional ROIs were manually segmented by a radiologist with more than 10 years of experience in GC diagnosis. The first ROI (ROI-1) was delineated on the tumor in the slice with the maximum tumor lesion. The second ROI (ROI-2) was delineated on the region of No.3 station LNs around the lesser curvature of stomach. ROI segmentation was performed using ITK-SNAP software (version 2.2.0; www.itksnap.org) on the venous phase CT images with axial view (see Additional file 1: A1 for detail).

### Extraction of radiomic features

Two feature groups were extracted from two ROIs, with each group containing 273 features [41, 42]. These features were divided into 4 categories: (a) shape and size features, (b) gray intensity features, (c) texture features, and (d) wavelet features. The feature extraction was implemented using MATLAB (version 2014a; Mathworks, Natick, MA, USA). Radiomic features of all patients were standardized by the z-score method, based on the parameters calculated from the training cohort. More information about the radiomic feature extraction is shown in Additional file 1: A2.

### Radiomic signature construction

Radiomic feature selection and signature building were performed in the training cohort for ROI-1 and ROI-2, respectively. More details are described as follows. In order to avoid model over-fitting and improve performance, feature selection was performed to match the sample size (Additional file 1: A3).

First, the minimum redundancy maximum relevance algorithm (mRMR) ranked each feature based on its relevance to LN metastasis status, and the ranking process was able to consider the redundancy of these features at the same time [43]. Since the number of predictors should be kept within 1/10–1/3 of the size of the group that contains the smallest cases in the training cohort (LN metastasis-positive group, n = 22) [44], the number of potential features was limited to 7 or less in this study.

Second, five-fold cross-validation was performed multiple times on the training cohort to find the optimal number of features with the best performance based on ranked features. Then a radiomic signature (RS1) reflecting phenotype of ROI-1 and a radiomic signature (RS2) reflecting phenotype of ROI-2, were built as independent predictors of LN metastasis using selected features, respectively. It should be noticed that the feature selection and radiomic signatures construction were implemented based on training cohort alone. For each radiomic signature, the signature score was calculated to reflect the risk of LN metastasis. The predictive performance of the radiomic signatures were quantitatively tested using the area under the receiver operator characteristic (ROC) curve in both the training and testing cohorts.

### Construction and evaluation of nomogram

Univariate analysis and multivariate analysis were used to screen out significant clinical risk factors. For univariate analysis, continuous variables were assessed using independent t-test or Mann–Whitney U test for differences between different groups, and categorical variables were assessed by Chi-squared test. A two-sided *P* value < 0.05 was used to indicate statistical significance. As for multivariate analysis, we performed multivariate logistic regression to screen out key factors. Furthermore, multivariate logistic regression was used to merge two radiomic signatures and clinical risk factors into a nomogram. Similarly, the building of radiomic nomogram was conducted based on training cohort alone. For comparison, we construct two more models which combine clinical risk factors with RS1and RS2, respectively. After that, the calibration curves and Hosmer–Lemeshow test were used to assess the goodness-of-fit of the nomogram, and the AUC was used to quantify its predictive performance. For assessing overfitting, DeLong test was adapted to compare AUCs between training and testing cohorts. Moreover, we used net reclassification index (NRI) to compare the performance between nomogram and clinical risk factors, and quantify the improvement in predictive performance.

Furthermore, a stratified analysis was used to evaluate the influence of clinical factors to the nomogram. In addition, we performed a subgroup analysis to evaluate the additional value of the nomogram in the CT-reported LN metastasis-negative (CT-LNM0) subgroup. Since the number of metastasis in No.4 station LNs (left greater curvature) ranked only behind No.3 station LNs (Additional file 1: Table S2), we further validated our nomogram on No.4 station LNs.

Finally, to estimate the clinical utility of the nomogram, decision curve analysis (DCA) was performed by calculating the net benefits using a range of threshold probabilities.

## Results

### Clinical characteristics

Table 1 summarizes the patients’ clinical risk factors in both the training and testing cohorts. There is no significant difference in the probability of LN metastasis between the two cohorts (*P* = 0.384). Univariable analysis showed that CT-reported LN metastasis status from the radiologist were significantly correlated with pathological LN metastasis status (*P* < 0.05), while CA125 was significantly correlated with LN metastasis status only in the training cohort and tumor infiltration depth in the testing cohort. After multivariable analysis we chose the CT-reported LN metastasis status to predict LN metastasis.

**Table 1:** Characteristics of patients in the training and testing cohorts

Characteristic	Training cohort		*P*-value	Testing cohort		*P*-value
	LNM (+)	LNM (−)		LNM (+)	LNM (−)	
Sex, No. (%)			0.451			0.366
Male	16 (72.73)	37 (63.79)		11 (64.71)	49 (79.03)	
Female	6 (27.27)	21 (36.21)		6 (35.29)	13 (20.97)	
Age, mean ± SD, years	59.82 ± 11.93	62.52 ± 10.69	0.276	58.82 ± 8.83	62.60 ± 9.86	0.173
CEA, No, (%)			0.114			0.204
Median (IQR)	2.19 (1.44–2.54)	1.31 (1.02–2.44)		2.46 (1.50–3.10)	1.86 (1.14–2.76)	
Normal	22 (100.00)	55 (94.83)		17 (100.00)	59 (95.16)	
Abnormal	0 (0.00)	3 (5.17)		0 (0.00)	3 (4.84)	
CA19-9, No, (%)			0.171			0.867
Median (IQR)	11.95 (8.22–16.46)	8.97 (6.24–15.59)		12.30 (7.12–16.11)	9.99 (6.56–15.81)	
Normal	21 (95.45)	57 (98.28)		17 (100.00)	60 (96.77)	
Abnormal	1 (4.55)	1 (1.72)		0 (0.00)	2 (3.23)	
CA125, No, (%)			0.035*			0.308
Median (IQR)	14.18 (8.70–22.66)	8.81 (5.43–16.04)		10.25 (8.69–13.10)	8.85 (4.95–15.69)	
Normal	20 (90.91)	57 (98.28)		16 (94.12)	60 (96.77)	
Abnormal	2 (9.09)	1 (1.72)		1 (5.88)	2 (3.23)	
Pathologic grade, No, (%)			0.349			0.410
Low grade	12 (54.55)	22 (37.93)		6 (35.29)	20 (32.26)	
Median grade	9 (40.91)	34 (58.62)		11 (64.71)	36 (58.06)	
High grade	1 (4.54)	2 (3.45)		0 (0.00)	6 (9.68)	
CT-reported LNM status, No, (%)			< 0.001*			< 0.001*
LNM0	12 (54.55)	54 (93.10)		11 (64.71)	58 (93.55)	
LNM1	10 (45.45)	4 (6.90)		6 (35.29)	4 (6.45)	
Tumor infiltration depth, No, (%)			0.181			< 0.001*
T1a	4 (18.18)	20 (33.48)		0 (0.00)	20 (32.26)	
T1b	9 (40.91)	25 (43.10)		4 (23.53)	31 (50.00)	
T2	9 (40.91)	13 (22.41)		13 (76.47)	11 (17.74)	

*P*-value was derived from the univariable association analyses between each characteristic and LNM status; For univariate analysis, independent *t* test or Mann–Whitney U test were used for continuous variables and Chi-squared test for categorical variables. *P value < 0.05; LNM0 refers to CT-reported LNM-negative; LNM1 refers to CT-reported LNM-positive

*LNM* lymph node metastasis, *SD* standard deviation, *CEA* carcinoembryonic antigen, *CA19-9* carbohydrate antigen 19-9, *CA125* cancer antigen 125, *CT* computed tomography

### Establishment of radiomic signature

**Fig. 2:**
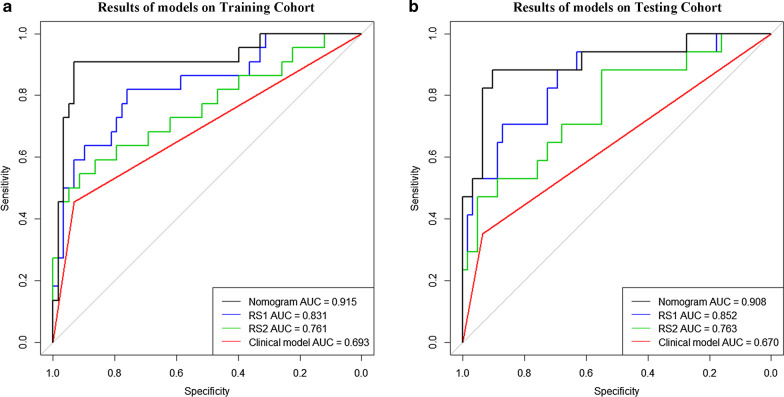
Performance of nomogram, signatures and CT-reported metastasis LN status in training and testing cohorts. *AUC* area under the curve. The black line represents the result of the nomogram. The blue line represents the result of the radiomic signature-1. The green line represents the result of the radiomic signature-2. The red line represents the result of the CT-reported metastasis LN status. *Abbreviations RS1* Radiomic signature 1, *RS2* radiomic signature 2

During the feature selection, mRMR selected top 10 radiomic features from ROI-1 and top 10 radiomic features from ROI-2 in the training cohort, respectively. As shown in Additional file 1: Figure S1 and Table S3, the cross-validation reserved 4 features from ROI-1 and 2 features from ROI-2. The heatmaps of these features and unsupervised cluster partitioning are shown in Additional file 1: Figure S2. Significant association was found between these features and LN metastasis status. Two radiomic signatures were built using linear combination of these radiomic features (4 features from ROI-1 for RS1 and 2 features from ROI-2 for RS2), and the signature score calculation are presented in Additional file 1: A4. As shown in Fig. 2 and Table 2, both of the two radiomic signatures showed significant predictive ability of LN metastasis in training cohort (AUC of RS1: 0.831, 95% confidence interval [CI] 0.725–0.937, and AUC of RS2: 0.761, 95% CI 0.629–0.893) and testing cohort (AUC of RS1: 0.852, 95% CI 0.742–0.962, and AUC of RS2: 0.763, 95% CI 0.626–0.900).

**Table 2:** Performance evaluation of models

Cohort	Models	TP	TN	FN	FP	Acc	Sen	Spe	PPV	NPV	AUC (95% CI)
Training cohort	R1	18	44	4	14	0.775	0.818	0.759	0.563	0.917	0.831 (0.725–0.937)
	R2	14	46	8	12	0.750	0.636	0.793	0.538	0.852	0.761 (0.629–0.893)
	CTR	10	54	12	4	0.800	0.455	0.931	0.714	0.818	0.693 (0.581–0.804)
	R1 + CTR	16	51	6	7	0.837	0.727	0.879	0.695	0.895	0.869 (0.789–0.949)
	R2 + CTR	16	46	6	12	0.775	0.727	0.793	0.571	0.885	0.814 (0.704–0.925)
	Nomogram	20	54	2	4	0.925	0.909	0.931	0.833	0.964	0.915 (0.832–0.998)
Testing cohort	R1	12	54	5	8	0.835	0.706	0.871	0.600	0.915	0.852 (0.742–0.962)
	R2	12	42	5	20	0.684	0.706	0.677	0.375	0.894	0.763 (0.626–0.900)
	CTR	6	58	11	4	0.810	0.353	0.935	0.600	0.841	0.644 (0.523–0.765)
	R1 + CTR	14	48	3	14	0.784	0.823	0.774	0.500	0.941	0.863 (0.772–0.954)
	R2 + CTR	14	45	3	17	0.747	0.824	0.726	0.452	0.938	0.753 (0.618–0.889)
	Nomogram	15	56	2	6	0.899	0.882	0.903	0.714	0.966	0.908 (0.814–1.000)

*R1* radiomic signature-1, *R2* radiomic signature-2, *CTR* CT-reported LN metastasis status, *TP* true positive, *TN* true negative, *FN* false negative, *FP* false positive, *Acc* accuracy, *Sen* sensitivity, *Spe* specificity, *PPV* positive predictive value, *NPV* negative predictive value, *AUC* area under curve, *CI* confidence interval

### Construction of nomogram

**Fig. 3:**
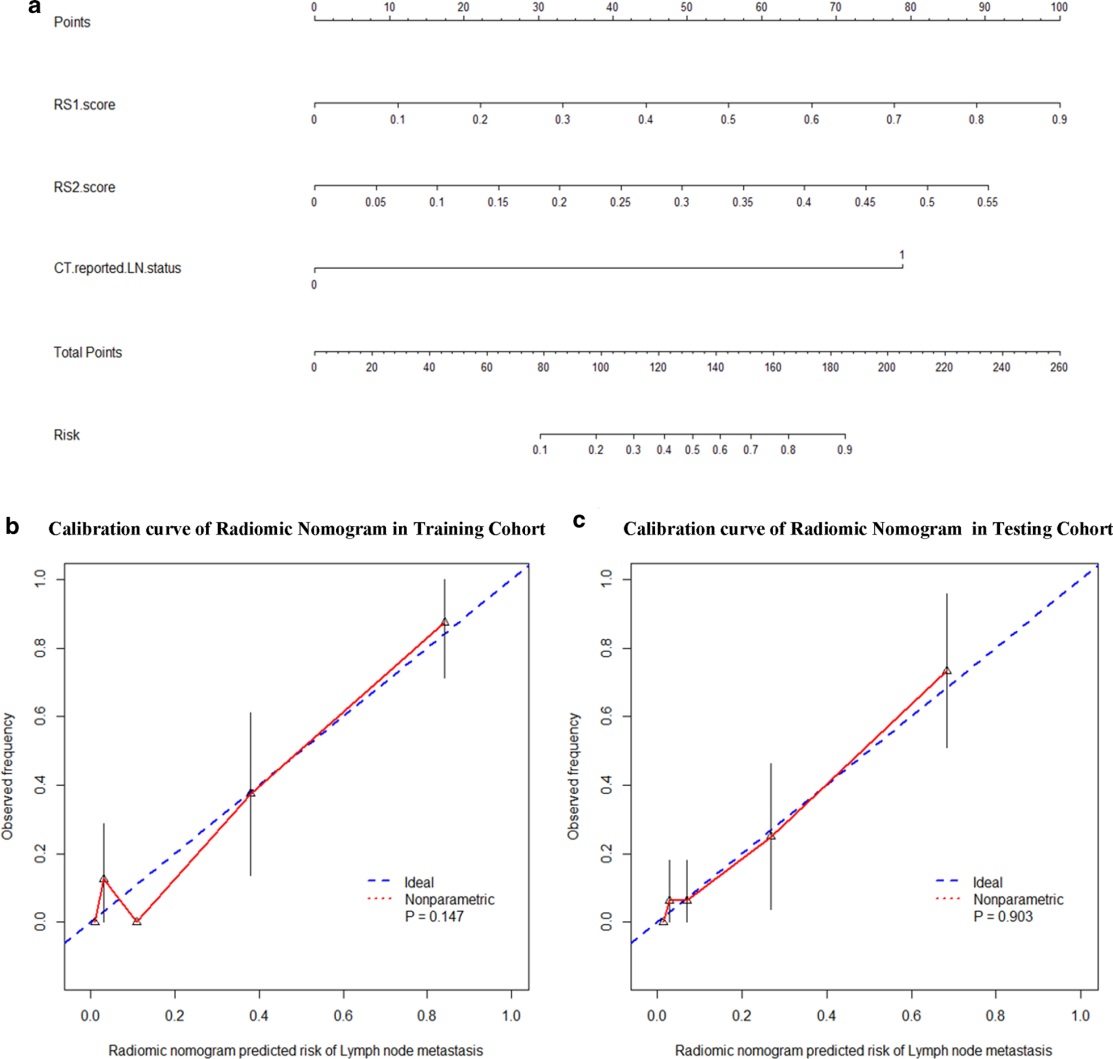
Nomogram and calibration curves. **a** Nomogram for the prediction of lymph node metastasis. **b** Calibration curves of the nomogram in the training cohort and **c** testing cohort. Calibration curves depict the calibration of the nomogram in terms of agreement between the predicted risk of lymph node metastasis and observed outcomes. The 45-degree blue dotted lines represent perfect prediction, and the pink lines represent the predictive performance of the nomogram. The closer the dotted line fit to the ideal line, the better the predictive accuracy of the nomogram. *LN* lymph node, *CT* computed tomography. *Abbreviation RS1* Radiomic signature 1, *RS2* radiomic signature 2

During the multivariate logistic regression analysis, the two radiomic signatures and one clinical risk factor (CT-reported LN metastasis status, CTR) were identified as independent predictors of LN metastasis in T1-2 GC patients (Additional file 1: Table S4). An individualized nomogram was built using the regression method to predict the LN metastasis probability (Fig. 3a).

### Evaluation of nomogram

As shown in Fig. 2 and Table 2, our nomogram reached an AUC of 0.915 (95% CI 0.832–0.998) in the training cohort and an AUC of 0.908 (95% CI 0.814–1.000) in the testing cohort, which were better than CTR, RS1, RS2, RS1 + CTR, and RS2 + CTR. The NRI also demonstrated that the nomogram had better predictive ability than the CT-reported LN metastasis status in the training cohort (NRI = 0.339, *P* < 0.001) and testing cohort (NRI = 0.301, *P* < 0.001). The DeLong test revealed that difference was not significant between AUCs of our nomogram in training and testing cohorts (*P* = 0.908), further indicating the robust of our nomogram. As shown in Fig. 3b, c, the calibration curves of the nomogram demonstrates a good fitness of nomogram in both the training and testing cohorts. The Hosmer–Lemeshow test also showed good performance of our nomogram in the training cohort (*P* = 0.147) and testing cohort (*P* = 0.903).

**Fig. 4:**
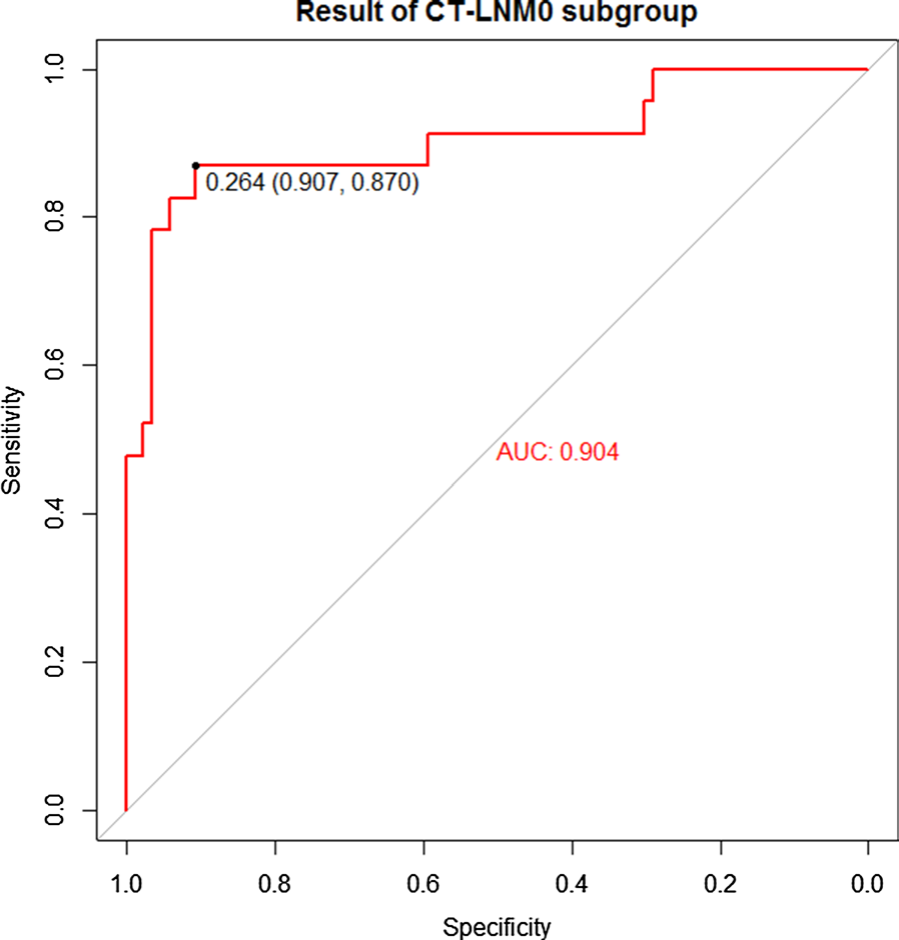
The result of comparative experiments in the CT-reported LN metastasis-negative subgroup. The panel shows the ROC curve analysis for the nomogram in the CT-LNM0 subgroup. *Abbreviations CT* computed tomography, *LN* lymph node, *ROC* receiver operator characteristic, *AUC* area under the curve

Notably, the subgroup analysis showed that our nomogram had a good discriminatory ability in the CT-LNM0 subgroup (n = 109, AUC 0.904; 95% CI 0.816–0.993; Fig. 4).

We also implemented stratified analysis, more details were presented in Additional file 1: A5 and Figure S3. The results showed that our nomogram worked well in gender, age, pathologic grade and tumor infiltration depth subsets (DeLong test, *P* > 0.05).

Moreover, we selected 9 patients with LN metastasis and 11 patients with non-LN metastasis at No.4 station as a validation set to further validate our nomogram. Interestingly, our nomogram also showed a good performance on this station (AUC 0.824; 95% CI 0.517–1; Additional file 1: Figure S4).

**Fig. 5:**
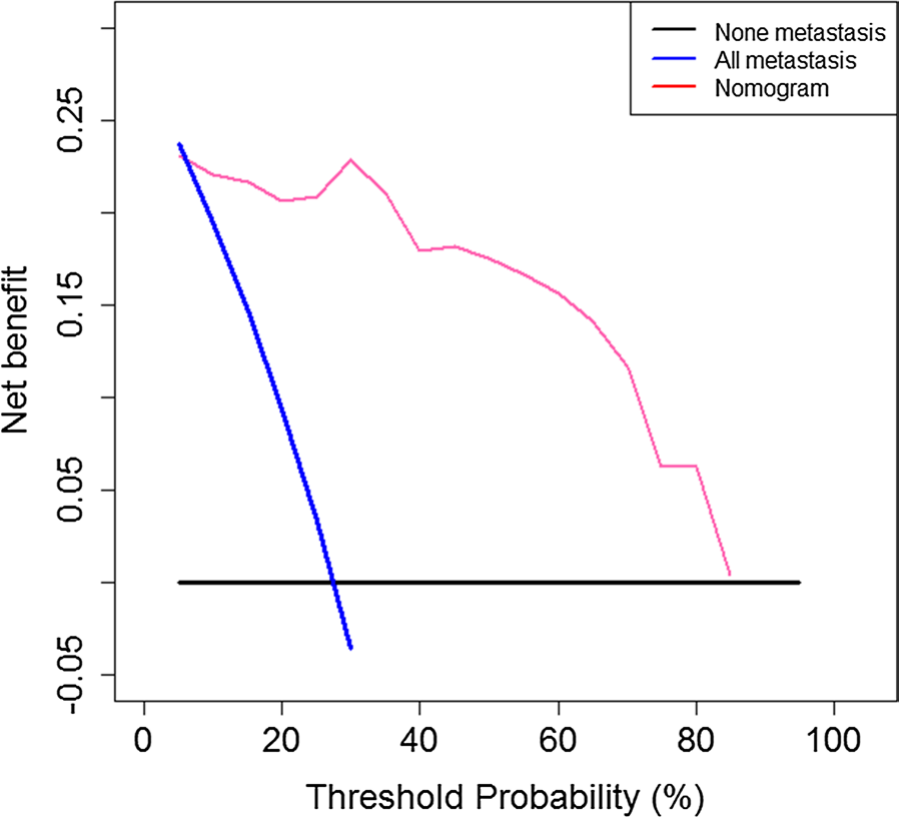
Decision curve analysis for the nomogram. The y-axis represents the net benefit, and the pink line represents the nomogram. The blue line represents the hypothesis that all patients had lymph node (LN) metastases, and the black line represents the hypothesis that no patients had LN metastases. The x-axis represents the threshold probability. The threshold probability is where the expected benefit of treatment is equal to the expected benefit of avoiding treatment. The decision curves in the training cohort showed that if the threshold probability is between 0 and 0.85, using the nomogram to predict LN metastases adds more benefit than treating either all or no patients

The decision curve of the nomogram is presented in Fig. 5. With a threshold of 0 to 0.85, patients using nomogram will have more diagnostic benefits than all-metastasis or none-metastasis strategies.

## Discussion

In this study, an easy-to-use radiomic nomogram was established to identify LN metastasis of T1-2 GC preoperatively. The nomogram, incorporating two radiomic signatures and CT-reported LN metastasis status, showed the best discrimination ability of LN metastasis in both the training and testing cohorts. The nomogram could assist the formulation of clinical treatment scheme.

Although the lymphatic system around the stomach is very complex [45], previous researches showed that the incidence rate of LN metastasis of EGC in No.3 station was the highest (No.3, 11.6%; No.1, 2.5%; No.2, 4.8%; No.4, 6.5%; No.5, 0.5%; No.6, 7.6%, respectively) (Additional file 1: Table S5) [38–40]. Therefore, we developed and validated a radiomics nomogram by integrating radiomics characteristics of No.3 LNs and primary tumors to better predict preoperative LNM in T1-2 GC patients.

We analyzed the radiomic features in the two significant radiomic signatures. The radiomic features used in RS1 included: (1) ‘X1_fos_skewness’ describes the shape of a probability distribution of the voxel intensity histogram, and reflects the distribution symmetry. (2) ‘X0_fos_variance’ measures the spread of intensity distribution about the mean value, and reflects the uniformity of distribution. (3) ‘X3_fos_root_mean_square’ is the root mean square of the voxels intensity value. (4) High ‘X1_GLCM_dissimilarity’ means there is a great disparity in intensity value among neighboring voxels. These radiomic features might quantify intratumor heterogeneity, and thus could predict the invasiveness of the tumor and the probability of LN metastasis [46]. The final selected radiomic features of lymph nodes consisted of: (1) ‘X1_GLRLM_energy’ measures of the magnitude of voxel values in an image describes the overall density of the lymph volume, (2) High ‘X1_GLCM_cluster_prominence’ implies more asymmetry. These radiomic features might indicate the high image intensity and heterogeneity in the No.3 station LN region, and thus the sign of LN metastasis. We have showed two examples of patients with and without LN metastasis (Additional file 1: A6 and Figure S5). The CT images also demonstrated that higher heterogeneity of the primary tumor and No.3 LN region leaded to higher probability of LN metastasis.

In this study, CT-reported LN metastasis status from the radiologist was significantly correlated with LN metastasis in univariable analysis. This subjective judgement was also included in our nomogram. We also found that CA125 was significantly associated with LN metastasis in the training cohort (*P* = 0.035), but had no significance in the testing cohort. This may be caused by the relatively small sample size and baseline deviation. Moreover, the positive rate of CA125 was very low in EGC [47].

We conducted some stratified analysis, the results showed that the performance of our nomogram was not affected by gender, age, pathologic grade and tumor infiltration depth factors. In addition, we tested the correlations between the radiomic features and clinical risk factors using Pearson correlation analysis (Additional file 1: Figure S6). There was no correlation between radiomic features and clinical risk factors, which pointed that the radiomic features might be a good supplement to clinical factors. The good performance of our nomogram in CT-LNM0 subgroup also demonstrated the additional value of the nomogram to the radiologists.

More interestingly, the nomogram trained from phenotype of No.3 station LNs also showed a positive role in predicting LN metastasis in No.4 station LNs. This finding indicated that the radiomic signature from the LN region did reflect the early change of phenotype of LNs. Thus, our nomogram may be used in other stations of LNs.

There are some limitations in this study. Firstly, the relatively small sample size of this study. Secondly, the lack of the external validation. Thirdly, the presence of lymphatic invasion and LN micrometastasis have also been considered as important risk factors for LN metastasis in EGC [48–50], however, these factors were not routinely collected in our center. Fourth, given the use of manual segmentation, the radiomic features reproducibility should be further evaluated. Finally, cases with invisible lesions on CT images were excluded, so some patients could not use the nomogram. These problems need to be further studied.

## Conclusions

In summary, the nomogram received favorable predictive accuracy in predicting No.3 LNM in T1-2 GC, and the nomogram showed positive role in predicting LNM in No.4 LNs. The radiomics nomogram may assist the formulation of clinical treatment scheme.

## Supplementary Information

**Additional file 1:** Supplementary methods, supplementary tables, and supplementary figures.

## Data Availability

The datasets and code used and analyzed during the current study are available from the corresponding author on reasonable request (13,913,433,095@163.com).

## Abbreviations

GC: Gastric cancer
LN: Lymph node
LNM: Lymph node metastasis
CT: Computed tomography
AUC: Area under the receiver operator characteristic curve
CI: Confidence interval
EGC: Early gastric cancer
ROI: Region of interest
mRMR: Minimum redundancy maximum relevance algorithm
RS: Radiomic signature
ROC: Receiver operator characteristic
NRI: Net reclassification index
CT-LNM0: CT-reported LN metastasis-negative
DCA: Decision curve analysis
