# Supplementary material for: Integrating No.3 lymph nodes and primary tumor radiomics to predict lymph node metastasis in T1-2 gastric cancer
Source: BMC Med Imaging. 2021 Mar 23;21:58. doi: 10.1186/s12880-021-00587-3 (PMC7989204; doi:10.1186/s12880-021-00587-3)
Supplement: Supplementary file 1 — Additional file 1: Supplementary methods, supplementary tables, and supplementary figures. [file 12880_2021_587_MOESM1_ESM.docx]

**Supplementary material**

**A1. ROI segmentation method**

We delineated three regions of interest (ROIs) to cover the region of tumor, No.3 lymph nodes (LNs), and No.4 LNs, respectively. The first ROI (ROI-1) was delineated on the tumor in the slice with the largest tumor area. The second ROI (ROI-2) was delineated on the region of No.3 LNs around the lesser curvature of stomach. The third ROI (ROI-3) was delineated on the region of No.4 LNs around the greater curvature of stomach. Note that ROI-1 and ROI-2 were delineated on all patients, but ROI-3 was only delineated on 20 patients. The detailed delineation methods are shown as follows.

ROI-1 (tumor region): select a slice with the largest tumor area and delineate a ROI to cover the margin of the tumor on this slice.

ROI-2 (No.3 LNs region): Delineate the ROI in the lesser curvature of the stomach. 1) Delineate the largest region between the first branch of the ascending branch of the LGA and the second branch of the RGA [1]. 2) Avoid perigastric vessels during the delineation.

ROI-3 (No.4 LNs region): Delineate the ROI in the greater curvature of the stomach. 1) Delineate the larges region along the short gastric arteries, the left gastroepiploic artery, and the 2nd branch and distal part of the right gastroepiploic artery [1]. 2) Avoid perigastric vessels during the delineation.

[1] Japanese Gastric Cancer Association (2011) Japanese classification of gastric carcinoma: 3rd English edition. *Gastric Cancer* 14:101-112.

**A2.** **CT radiomic feature extraction**

A filtering process was performed to implement image smoothing before computed tomography (CT) feature extraction. Separable filtering was used to avoid the multi-dimensional convolution. The convolution was performed with a low-/high-pass “Coiflet 1” wavelet filter along the x-/y-direction, separately. Consider *L* and *H* to be low-pass and high-pass functions, respectively, *X* to be the original CT image, and the filtered results of *X* to be labelled as$X_{LL},X_{LH}X_{HL,}X_{HH}$. That is, four new images were obtained by filtering the original image in two directions (x, y).

After filtering, a total of 273 quantitative features were extracted from each ROI of the original image and its corresponding filtered results, including the features from the categories of histogram, shape, gray-level co-occurrence matrix (GLCM), and gray-level run-length matrix (GLRLM). The process of image filtering and feature extraction was performed using an in-house software implemented in MATLAB software (version 2014a; Mathworks, Natick, MA, USA).

**First-order statistical features**: First-order statistics described the distribution of voxel intensities within the CT image through commonly used and basic metrics. To analyze the spatial distribution of the pixels' hue matrix and extract static features of images, a fuzzy similitude matrix was defined. The matrix described the image's feature space. Seven first-order statistical features were used, including energy, entropy, skewness, kurtosis, mean, maximum, and minimum.

**Shape- and size-based features**: In this group of features, we included eight descriptors of the three-dimensional size and shape of the tumor region. They included surface area, volume, surface-to-volume ratio, maximum three-dimensional diameter, sphericity, spherical disproportion, and compactness 1 and 2.

**Statistics-based textural features**: Textural features are visual characteristics that reflect the homogeneity phenomenon of images and the arrangement of properties that change slowly or periodically on the body surface. Our textural features mainly included two typical matrices: the Gray-level co-occurrence matrix (GLCM) and the Gray-level run-length texture matrix (GLRLM). GLCM is the matrix function that describes the distance and angle of each pixel. By calculating the correlation between two gray levels with certain directions and distances, GLCM can reflect integrated information regarding the direction, interval, amplitude, and frequency of images. GLRLM can quantify gray level runs in an image. Gray level runs are defined as the lengths (number of consecutive pixels) that have the same gray-level value. We extracted 22 radiomic features from the GLCM and 14 features from the GLRLM. The radiomic features in the GLCM mainly consisted of energy, entropy, correlation, contrast, homogeneity, autocorrelation, mean, variance, dissimilarity, and angular second moment. The radiomic features in the GLRLM mainly consisted of features such as run length non-uniformity, short/long run emphasis, and Gray level non-uniformity.

**Wavelet features**: The undecimated three-dimensional (3D) wavelet transform was used to decompose the original image, which can be regarded as a preprocessing prior to feature extraction. By changing the ratio of high-frequency to low-frequency signal in images, wavelet transform increases the information of low-frequency signal. The size of each decomposition is equal to the original image, and each decomposition is shift invariant. For each decomposition, we computed the first-order statistics and textural features described above. This resulted in 212 features. In the end, we extracted 273 features for each of the CT image.

The six selected features were as follows:

1. **First Order Features**

X represents a three-dimensional image of the tumor, $N_{p}$ represents the total number of tumor pixels.

***X1_fos_skewness***: Skewness measures the asymmetry of the distribution of values about the mean value. Depending on where the tail is elongated and the mass of the distribution is concentrated, this value can be positive or negative.

skewness = $\frac{\mu_{3}}{\sigma^{3}}$ = $\frac{\frac{1}{N_{p}}\sum_{i=1}^{N_{p}} {(X(i)-\overline{X})}^{3}}{{\sqrt{\frac{1}{N_{p}}\sum_{i=1}^{N_{p}} {X(i)-\overline{X}}^{2}}}^{3}}$

where $\mu_{3}$ is the $3^{rd}$ central moment.

***X0_fos_variance***: Variance is the mean of the squared distances of each intensity value from the Mean value. This is a measure of the spread of the distribution about the mean. By definition, variance = σ^2^

variance = $\frac{1}{N_{p}}\sum_{i=1}^{N_{P}} {(X(i)-\overline{X})}^{2}$

***X3_fos_root_mean_square (RMS)***: RMS is the square-root of the mean of all the squared intensity values. It is another measure of the magnitude of the image values. This feature is volume-confounded, a larger value of C increases the effect of volume-confounding.

RMS = $\sqrt{\frac{1}{N_{p}}\sum_{i=1}^{N_{P}} {(X(i)+C)}^{2}}$

Here, C is an optional value, defined by voxelArrayShift, which shifts the intensities to prevent negative values in X. This ensures that voxels with the lowest gray values contribute the least to RMS, instead of voxels with gray level intensity closest to 0.

1. **Gray Level Co-occurrence Matrix (GLCM) Features**

A Gray Level Co-occurrence Matrix (GLCM) of size $N_{g}\times N_{g}$, describes the second-order joint probability function of an image region constrained by the mask and is defined as $P(i,j|\delta,\theta)$. The ${(i,j)}^{th}$ element of this matrix represents the number of times the combination of levels $i$ and$j$ occur in two pixels in the image, that are separated by a distance of $\delta$ pixels along angle $\theta$. The distance $\delta$ from the center voxel is defined as the distance according to the infinity norm. $\mu_{x}$ be the mean gray level intensity of px and defined as $\mu_{x}= \sum_{i=1}^{N_{g}} p_{x}(i)i$. $\mu_{y}$ be the mean gray level intensity of px and defined as $\mu_{y}= \sum_{j=1}^{N_{g}} p_{y}(j)j$.

***X1_GLCM_dissimilarity***: Dissimilarity is a measure of local intensity variation defined as the mean absolute difference between the neighboring pairs. A large value correlates with a greater disparity in intensity value among neighboring voxels.

dissimilarity = $\sum_{i=1}^{N_{g}} \sum_{j=1}^{N_{g}} p\left( i,j \right)|i-j|$

***X1_GLCM_cluster_prominence***: Cluster Prominence is a measure of the skewness and asymmetry of the GLCM. A higher value implies more asymmetry about the mean while a lower value indicates a peak near the mean value and less variation about the mean.

cluster prominence = $\sum_{i=1}^{N_{g}} \sum_{j=1}^{N_{g}} {(i+j-\mu_{x}-\mu_{y})}^{4}p(i,j)$

1. **Gray Level Run Length Matrix (GLRLM) Features**

A Gray Level Run Length Matrix (GLRLM) quantifies gray level runs, which are defined as the length in number of pixels, of consecutive pixels that have the same gray level value. In a gray level run length matrix $P(i,j|\theta)$, the ${(i,j)}^{th}$ element describes the number of runs with gray level $i$ and length $j$ occur in the image (ROI) along angle $\theta$.

***X1_GLRLM_energy***: Energy is a measure of the magnitude of voxel values in an image. A larger value implies a greater sum of the squares of these values.

energy = $\sum_{i=1}^{N_{p}} {(X\left( i \right)+C)}^{2}$

Here, C is optional value, defined by voxelArrayShift, which shifts the intensities to prevent negative values in X. This ensures that voxels with the lowest gray values contribute the least to Energy, instead of voxels with gray level intensity closest to 0.

**A3. Power calculation**

In our paper, the multiple logistic regression analysis was selected as the method of modeling the predictors of LN metastasis status. It is important to estimate the minimum sample size for training and validation for our study.

**1. Sample size evaluation of the training cohort:**

For the training sample size, Chalkidou and colleagues proposed that for linear models, like multiple regression, at least 10 to 15 observations per predictor variable were required to produce reasonably stable estimates [2]. In our study, there were four primary lesion features, two LN features, and one clinical risk factor used for the final model, indicating the sample size should be no less than 70 subjects. The training sample size in our study was 80.

**2. Sample size evaluation of the testing cohort:**

For the testing sample size, Shein-Chung Chow and colleagues [3] introduced a sample size estimation method for clinical research. According to their book, the sample size calculation to test whether the means of two groups are significantly different refers to the following formula.

Letting the two groups be $A$ and $B$, $\mu$ represents the mean in each group, with the hypotheses of interest being:

$$H_{0}:\mu_{A}-\mu_{B}=0$$

$$H_{1}:\mu_{A}-\mu_{B}\neq0$$

The sample size and power are calculated respectively:

$$N_{A}=\left( \frac{n_{A}+n_{B}}{n_{B}} \right)\left( \sigma\frac{z_{1-\alpha/2}+z_{1-\beta}}{\mu_{A}-\mu_{B}} \right)^{2}$$

$$N_{B}=\left( \frac{n_{A}+n_{B}}{n_{A}} \right)\left( \sigma\frac{z_{1-\alpha/2}+z_{1-\beta}}{\mu_{A}-\mu_{B}} \right)^{2}$$

$1-\beta=\Phi\left( z-z_{1-\alpha/2} \right)+\Phi\left( -z-z_{1-\alpha/2} \right)$, $z=\frac{\mu_{A}-\mu_{B}}{\sigma\sqrt{\frac{1}{n_{A}}+\frac{1}{n_{B}}}}$

where, $n$ is the sample size in the training group and $N$ is the sample size for the testing group, $\Phi$ is the standard Normal distribution function, $\alpha$ is the Type I error, $\beta$ is the Type II error, $1-\beta$ is the power, and $\sigma^{2}$ is the variance of the covariate.

In our study, the sample sizes in the training groups were $n_{A}=58$ and $n_{B}=22$ with means of $\mu_{A}=0.1252$ and $\mu_{B}=0.6699$, respectively, and with a standard deviation of $\sigma=0.3246$.

Therefore, the minimum numbers of testing samples were 17 and 7, respectively, in the two groups with the desired two-sided significance level of $\alpha$ = 0.05 and power of $1-\beta$ = 95%. In our study, the testing cohort included 62 and 17 patients in the two groups, respectively, which all exceeded the minimum required sample sizes.

[2] Chalkidou A, O'Doherty MJ, Marsden PK(2015) False Discovery Rates in PET and CT Studies with Texture Features: A Systematic Review. *PLoS One* 10: e124165.

[3] Chow S, Shao J, Wang H (2008) Sample Size Calculations in Clinical Research. 2nd Ed. Publisher location: Chapman &Hall; 2008.//CRC Biostatistics Series.

**A4. Radiomic signature score calculation formulas:**

Rad-score (ROI-1) = 21.9850 - 0.2117*X1_fos_skewness - 0.0007*X0_fos_variance

- 0.1920*X3_fos_root_mean_square – 0.8969*X1_GLCM_dissimilarity

Rad-score (ROI-2) = 0.0755 – 0.0002*X1_GLRLM_energy – 0.0166*X1_GLCM_cluster_prominence

**A5. Stratified analysis of radiomic nomogram:**

In order to test the generalization of our radiomic nomogram, we performed stratified analysis on the subsets of sex, age, pathologic grade and tumor infiltration depth. ROC curves were used to evaluate the nomogram performance on subsets and showed a great robustness of our radiomic nomogram (Supplementary Figure S3). Meanwhile, we used DeLong test to assess whether there is a significant difference between subsets, there is no significant difference among AUCs of subsets and overall cohort.

1. **Stratified analysis on sex:** Patients are divided into two subsets: male and female with AUCs of 0.885 and 0.951 (DeLong test *P* value: 0.3392 and 0.8277 compared with the result of overall cohort).
2. **Stratified analysis on age:** Patients are divided into two subsets: age < 60 and age >= 60 with AUCs of 0.863 and 0.962 (DeLong test *P* value: 0.6423 and 0.3220 compared with the result of overall cohort).
3. **Stratified analysis on pathologic grade:** Patients are divided into two subsets: low pathologic grade and median and high pathologic grade with AUCs of 0.850 and 0.950 (DeLong test *P* value: 0.6248 and 0.4467 compared with the result of overall cohort).
4. **Stratified analysis on tumor infiltration depth:** Patients are divided into two subsets: T1a and T1b infiltration depth and T2 infiltration depth with AUCs of 0.868 and 0.951 (DeLong test *P* value: 0.7381 and 0.5376 compared with the result of overall cohort).

**A6.** **The probability of the LN metastasis using nomogram:**

The basic principle of our nomogram is to calculate a nomogram points for each patient, which is the sum of its predictors’ point. The nomogram point corresponds to a probability of the LN metastasis. We added two examples, one patient with LN metastasis and the other without. Supplementary Figure S5 (A) shows two patients’ CT images, corresponding ROIs, radiomic signature scores, CT-reported LN status, predictor point, total points and risk. Take patient 1 as an example, the value of each predictor and the corresponding point in the nomogram are shown in Supplementary Figure S5 (B). The total point of the patient is 141 (6+56+79) based on the nomogram, and the probability of lymph node metastasis corresponds to about 60%.

**Supplementary Tables**

**Table S1.** Pathologic grade.

| Grade | Grade Definition |
| --- | --- |
| GX | Grade cannot be assessed |
| G1 (Low grade) | Well differentiated |
| G2 (Median grade) | Moderately differentiated |
| G3 (High grade) | Poorly differentiated, undifferentiated |

**Table S2.** Number of patients with metastatic lymph nodes according to the tumor infiltration and station.

| Lymph node station | No.1 | No.2 | No.3 | No.4 | No.5 | No.6 | No.7 |
| --- | --- | --- | --- | --- | --- | --- | --- |
| T1a (mucosa)  (n=44) | 0 | 0 | 6(13.6%) | 1(2.3%) | 0 | 0 | 0 |
| T1b (submucosa)  (n=69) | 0 | 0 | 12(17.3%) | 3(4.3%) | 0 | 2(2.9%) | 0 |
| T2 (musculairs propria)  (n=46) | 0 | 0 | 21(45.6%) | 5(10.8%) | 2(4.3%) | 2(4.3%) | 0 |

**Table S3.** Radiomic features.

| Region | Features | Group | Filters | *P*-value |
| --- | --- | --- | --- | --- |
| Primary tumor | X1_fos_skewness | Histogram | *X_LL_* | < 0.001*** |
|  | X0_fos_variance | Histogram | NA | < 0.001*** |
|  | X3_fos_root_mean_aquare | Histogram | *X_HL_* | < 0.001*** |
|  | X1_GLCM_dissimilarity | GLCM | *X_LL_* | < 0.001*** |
| Lymph node | X1_GLRLM_energy | GLRLM | *X_LL_* | < 0.001*** |
|  | X1_GLCM_cluster_prominence | GLCM | *X_LL_* | < 0.001*** |

NOTICE. *** denotes *P*-value < 0.001. Abbreviations: GLRLM: gray level run length matrix; GLCM: gray level co-occurrence matrix.

**Table S4.** Risk factors of Nomogram.

| **Variable** | **β** | **Adjusted OR (95% CI)** | ***P*-value** |
| --- | --- | --- | --- |
| Intercept | -5.4420 |  | < 0.001* |
| Radiomic signature1  (per 0.1 increase) | 0.4588 | 1.5822 (1.1717 – 2.2429) | 0.005* |
| Radiomic signature2  (per 0.1 increase) | 0.6787 | 1.9713 (1.1757 – 3.7322) | 0.019* |
| CT-reported LN metastasis status | 3.2574 | 25.9822 (4.6468 - >100) | < 0.001* |

NOTICE. * denotes p-value < 0.05. Abbreviations: LN, lymph node; OR, odds ratio; CI: confidence interval; CT, computed tomography.

**Table S5.** The frequency of involvement of each nodal station.

| Group of lymph node | [Incidence](#C:/Program%20Files%20(x86)/Youdao/Dict/6.3.69.8341/resultui/frame/javascript:void(0);) rate of involvement (%) | | | | | | |
| --- | --- | --- | --- | --- | --- | --- | --- |
|  | Gastric cancer | |  |  | Early stage gastric cancer | | |
| No.1 |  | 63.6 |  | |  | 2.5 |  |
| No.2 |  | 38.5 |  | |  | 4.8 |  |
| No.3 |  | 75.0 |  | |  | 11.6 |  |
| No.4 |  | 53.3 |  | |  | 6.5 |  |
| No.5 |  | 14.2 |  | |  | 0.5 |  |
| No.6 |  | 46.3 |  | |  | 7.6 |  |

**Supplementary Figures**


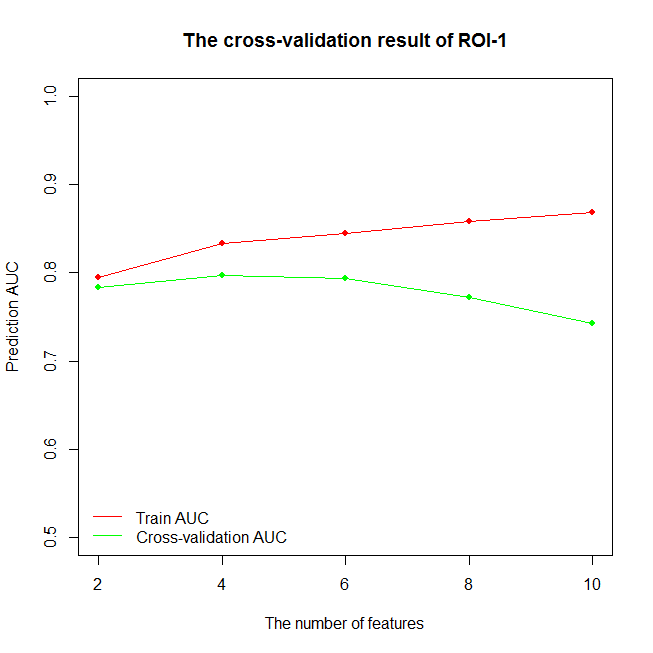

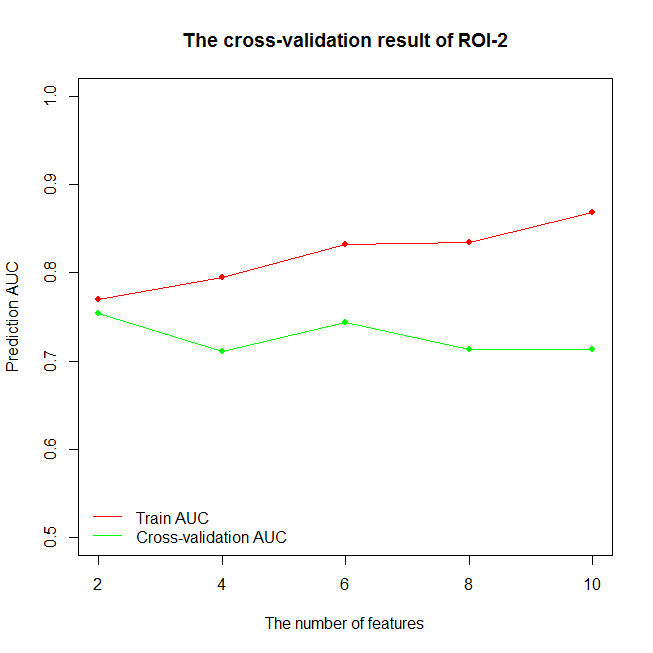


A

B

**Figure S1.** Results of cross-validation for selecting features from ROI-1 (A) and ROI-2 (B). The red line represents the result of the training set. The green line represents the result of the cross-validation.


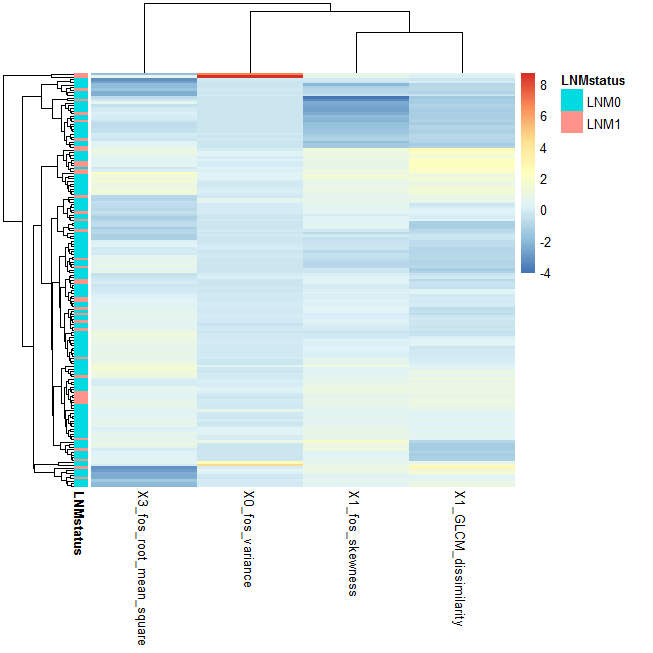

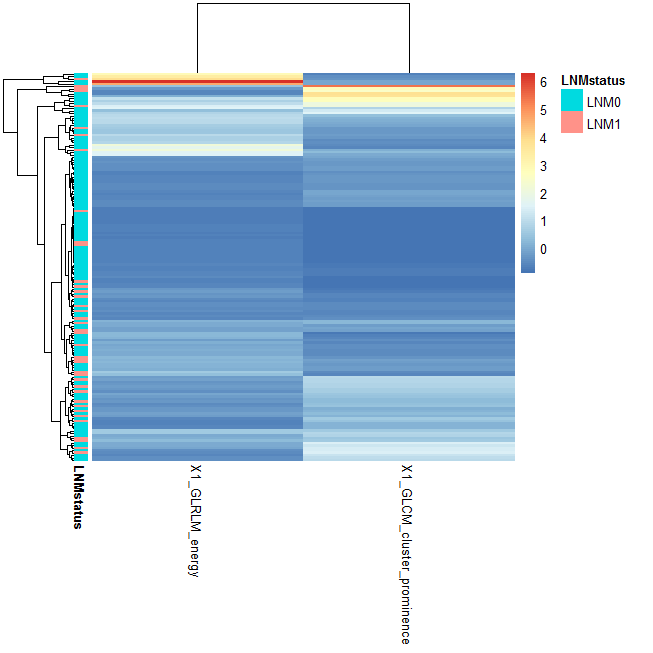


A

B

**Figure S2.** Heatmaps of radiomic features and all patients with unsupervised classification. (A) Result of training cohort. (B) Result of testing cohort.


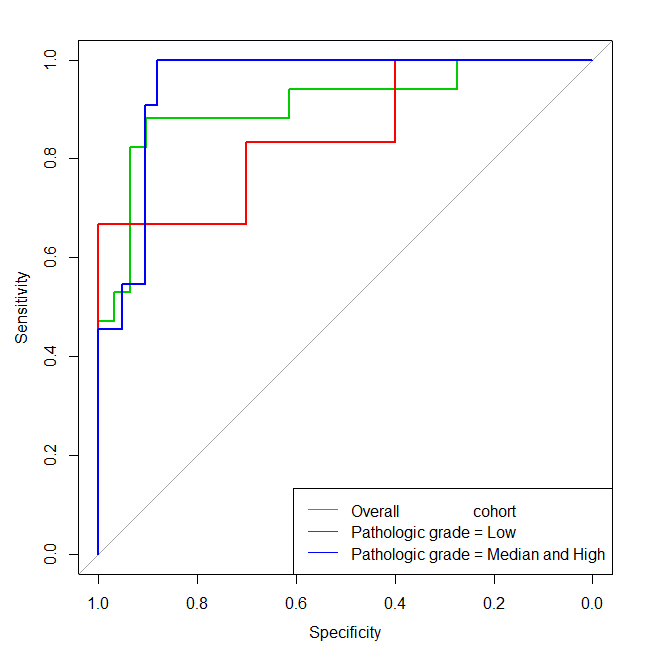

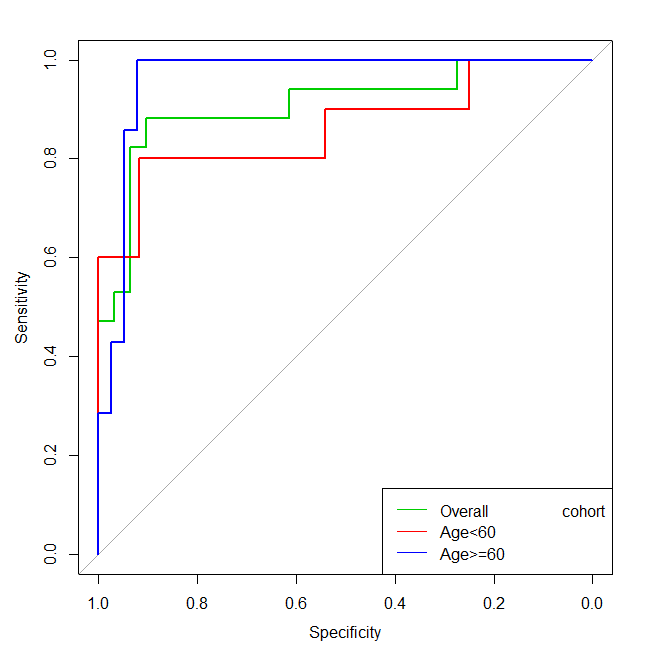

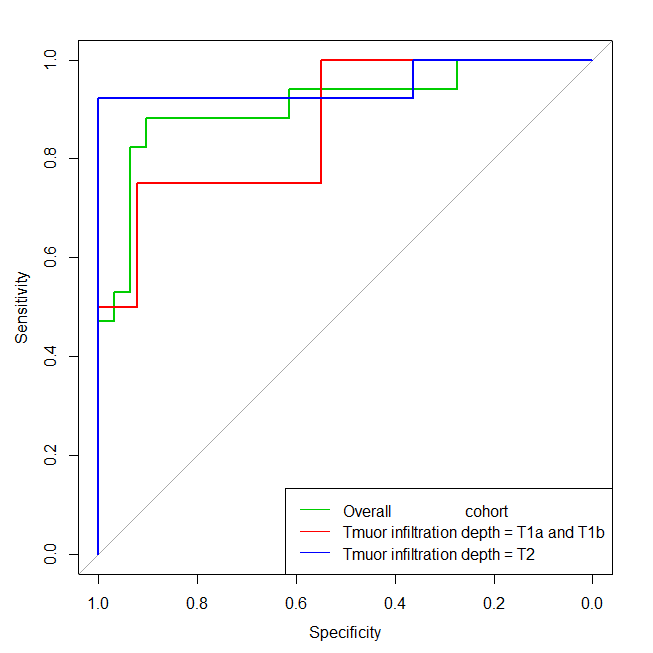

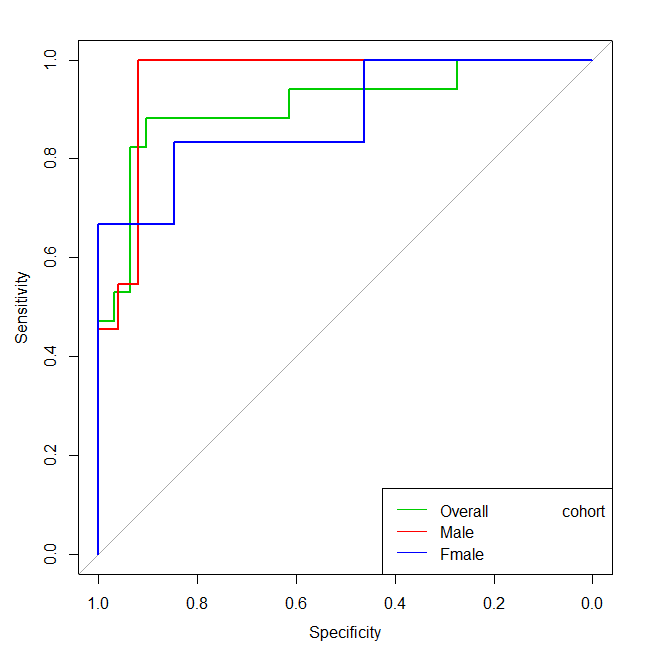


A

B

C

D

**Figure S3.** Nomogram performance for each subgroup stratified by (A) sex, (B) age, (C) pathologic grade, (D) tumor infiltration depth.


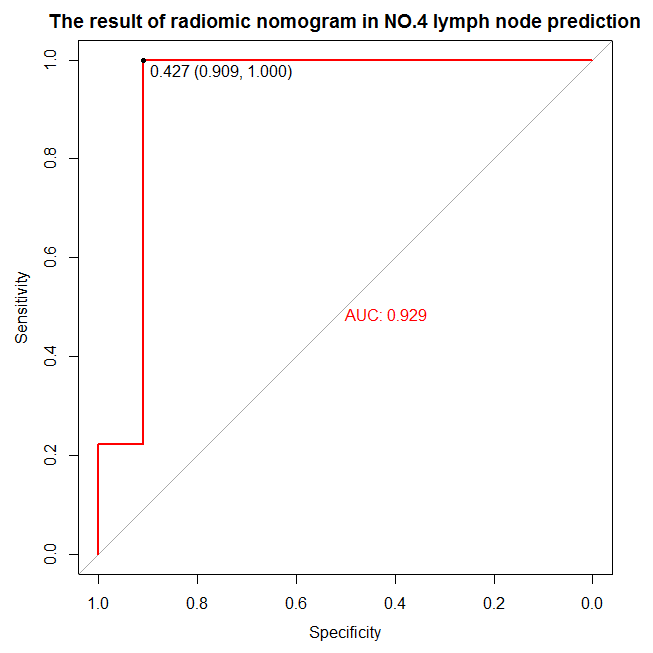


**Figure S4.** Performance evaluation of the nomogram in lymph node metastasis prediction at No.4.


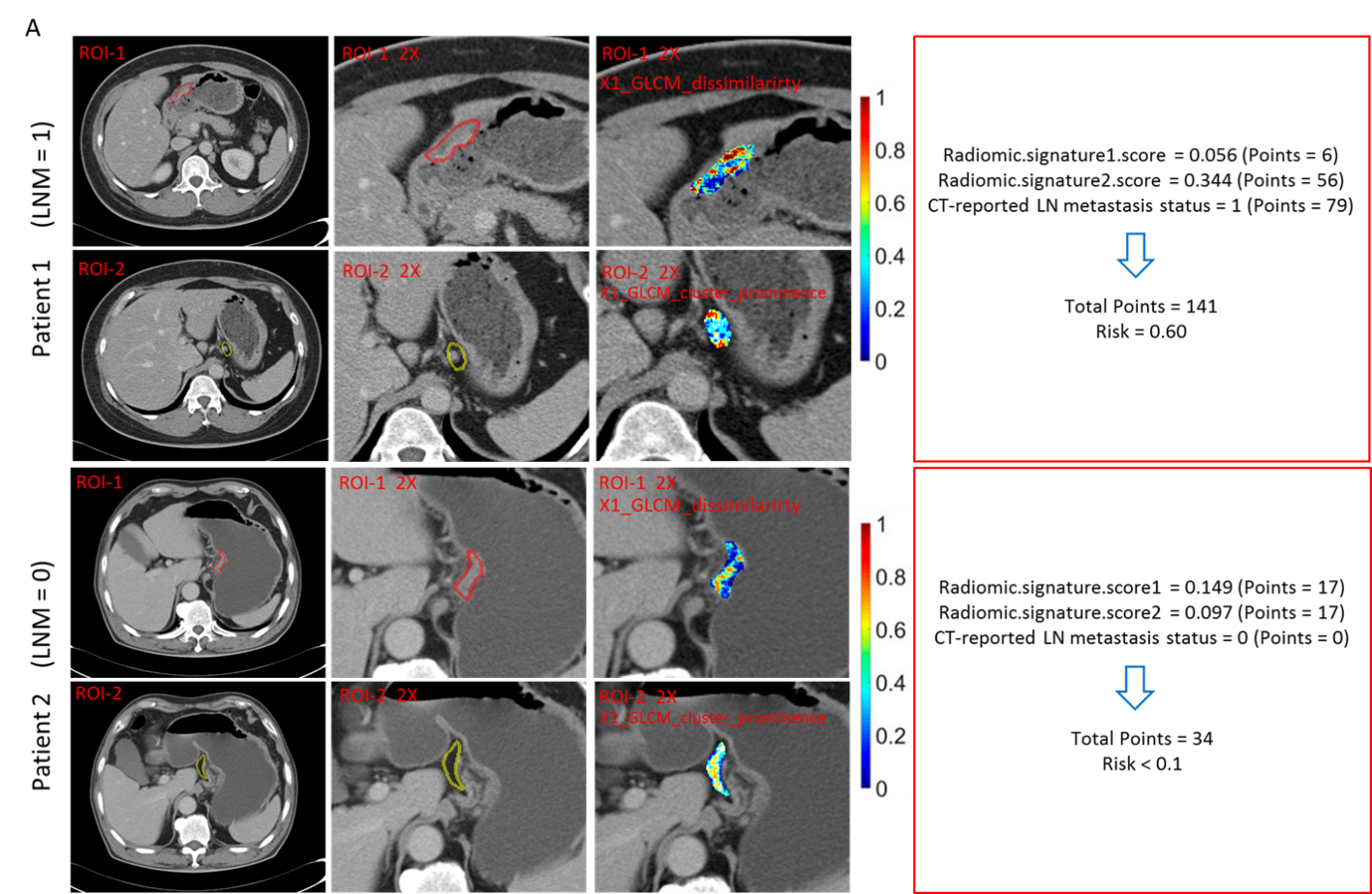


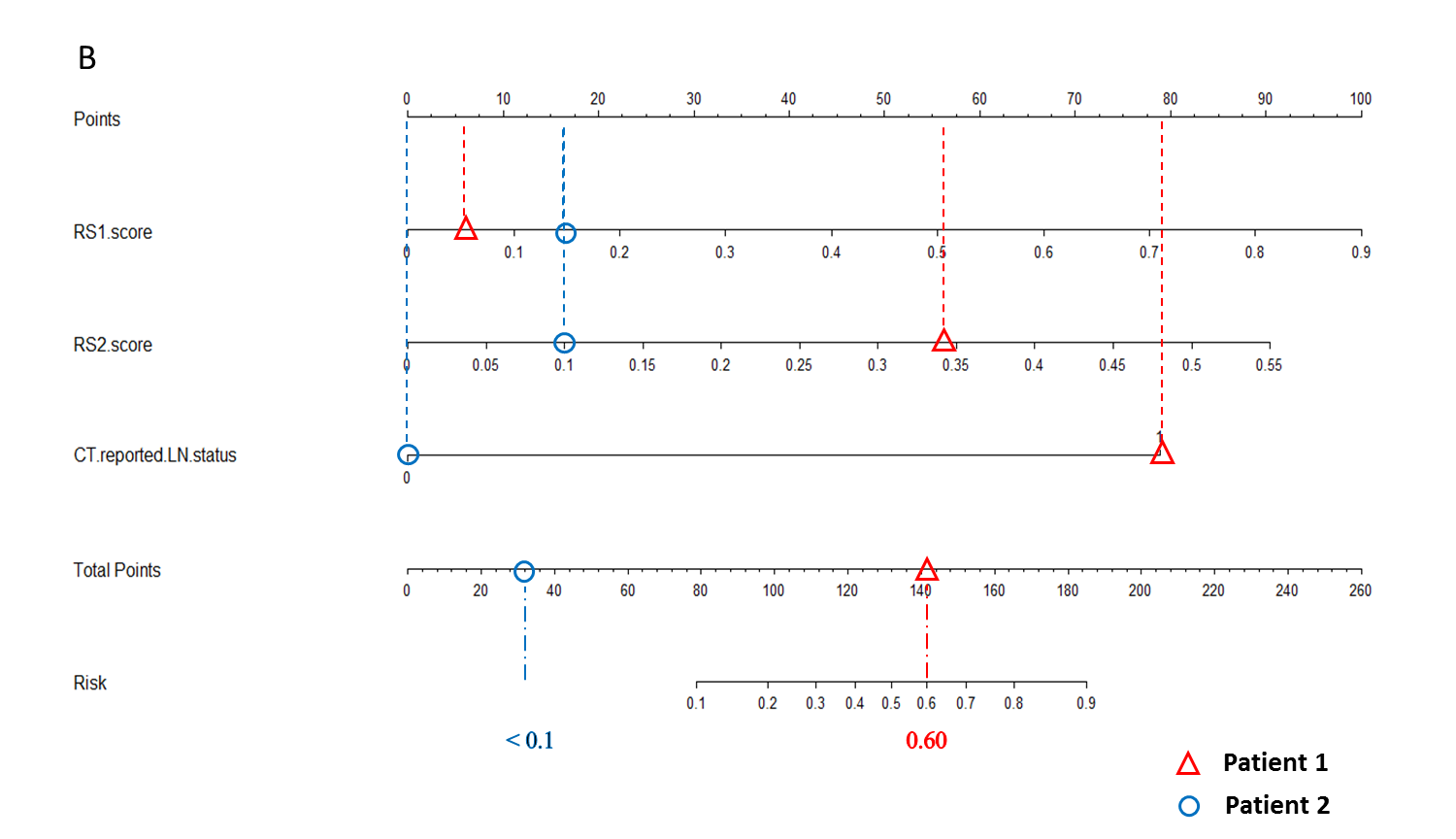


**Figure S5.** (A) CT images of two patients, corresponding ROIs and ROIs with computed features values overlaid on the same areas. ROI-1 means ROI of primary tumor, ROI-2 means ROI of NO.3 station LNs. 2X means that the picture was magnified twice. Patients’ radiomic signature scores, corresponding points, total points and the final risk are presented in red boxes. The texture features were computed per voxel by using a 3×3×3 patch centered at each voxel. (B) Nomogram with two examples of early stage gastric cancer patient. Abbreviations: RS1, Radiomic signature 1; RS2, Radiomic signature 2.


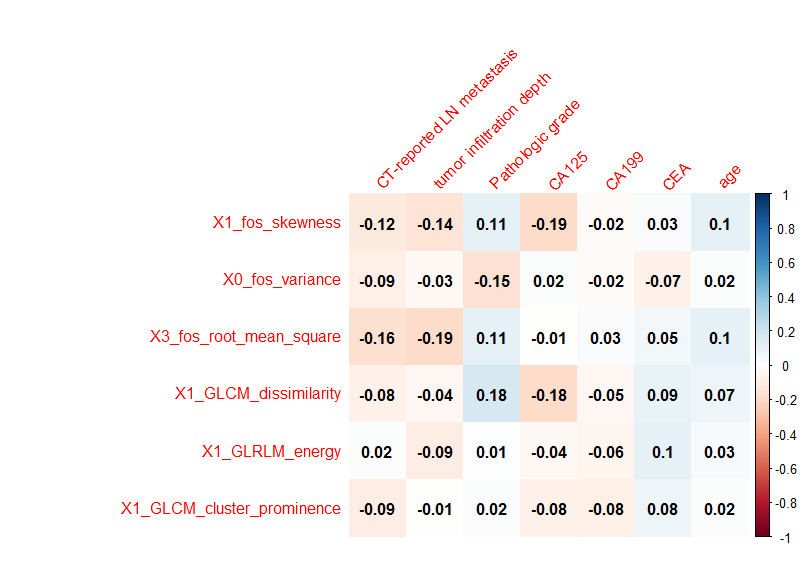


**Figure S6.** Pearson correlation result of radiomic features and clinical risk factors.
